# The impact of statins on aldosterone production in healthy adults: a randomized controlled study

**DOI:** 10.1210/jendso/bvag141

**Published:** 2026-06-30

**Authors:** Yan Emily Yuan, Lindsey M Porter, Andrea V Haas, Ezra S Hornik, Andrew W Koefoed, Rebecca Easly-Merski, Gillian Murray, Subrina Farah, Gail K Adler, Jonathan S Williams

**Affiliations:** Division of Endocrinology, Diabetes and Metabolism, Department of Medicine, Brigham and Women’s Hospital of Mass General Brigham, Harvard Medical School, Boston, MA 02115, USA; Division of Endocrinology, Diabetes and Metabolism, Department of Medicine, Brigham and Women’s Hospital of Mass General Brigham, Harvard Medical School, Boston, MA 02115, USA; Division of Endocrinology, Diabetes and Metabolism, Department of Medicine, Brigham and Women’s Hospital of Mass General Brigham, Harvard Medical School, Boston, MA 02115, USA; Division of Endocrinology, Diabetes and Metabolism, Department of Medicine, Brigham and Women’s Hospital of Mass General Brigham, Harvard Medical School, Boston, MA 02115, USA; Division of Endocrinology, Diabetes and Metabolism, Department of Medicine, Brigham and Women’s Hospital of Mass General Brigham, Harvard Medical School, Boston, MA 02115, USA; Division of Endocrinology, Diabetes and Metabolism, Department of Medicine, Brigham and Women’s Hospital of Mass General Brigham, Harvard Medical School, Boston, MA 02115, USA; Division of Endocrinology, Diabetes and Metabolism, Department of Medicine, Brigham and Women’s Hospital of Mass General Brigham, Harvard Medical School, Boston, MA 02115, USA; Division of Endocrinology, Diabetes and Metabolism, Department of Medicine, Brigham and Women’s Hospital of Mass General Brigham, Harvard Medical School, Boston, MA 02115, USA; Division of Endocrinology, Diabetes and Metabolism, Department of Medicine, Brigham and Women’s Hospital of Mass General Brigham, Harvard Medical School, Boston, MA 02115, USA; Division of Endocrinology, Diabetes and Metabolism, Department of Medicine, Brigham and Women’s Hospital of Mass General Brigham, Harvard Medical School, Boston, MA 02115, USA

**Keywords:** aldosterone, cardiovascular health, hyperlipidemia, pravastatin, simvastatin, statins

## Abstract

**Background:**

Statins lower atherosclerotic cholesterol, but the cardiovascular benefit from treatment may extend beyond lipid management. Our group previously described in an observational study that individuals on statin therapy had 33% lower aldosterone (ALDO) than individuals not on statins, and this effect was greater in individuals taking a lipophilic statin (eg, simvastatin) than a hydrophilic statin (eg, pravastatin). Further, studies in isolated rodent adrenal zona glomerulosa cells demonstrated that statins lowered ALDO, and again lipophilic statins had a greater effect. We, therefore, tested the hypothesis that treatment with simvastatin would result in lower than treatment with pravastatin or placebo in a randomized controlled trial.

**Methods:**

We conducted a 12-week, double-blind, randomized, placebo-controlled clinical trial among healthy adults with low-density lipoprotein cholesterol (LDL-C) > 70 mg/dL. Participants were randomized 1:1:1 to placebo, simvastatin 20 mg, or pravastatin 40 mg, stratified by sex. The study medication dose was doubled at Week 6 if the LDL-C decreased by less than 35% from screening values. We performed ALDO assessments at pretreatment, Week 6, and Week 12 posttreatment after 5 days on a controlled low-sodium diet.

**Results:**

Multivariate regression analysis controlling for sex, age, race, BMI, and pretreatment angiotensin II (AngII)-stimulated ALDO showed no significant differences in 12-week posttreatment AngII-stimulated ALDO between placebo and simvastatin (*P*, .56; primary endpoint) nor between simvastatin and pravastatin (*P*, .90; secondary endpoint).

**Conclusion:**

Among healthy individuals on a low-sodium diet, 12-week treatment with simvastatin or pravastatin did not alter ALDO responsiveness to AngII.

Cardiovascular disease (CVD) is the leading cause of death worldwide [1]. Modifying risk factors, such as the treatment of hypercholesterolemia, is a standardized part of CVD management [2, 3]. Since the first statin was approved for use in the United States in 1987, statins have become a mainstay in the management of hypercholesterolemia and have been shown to be effective in both primary and secondary prevention of CVD [3, 4]. In addition to the well-established role of statins to lower atherosclerotic lipids, the observed benefit of statins on cardiovascular health likely also arises from other pleiotropic mechanisms, including improvements in blood pressure, reno-protective benefits, and improved cardiac function in heart failure [5-12].

Aldosterone (ALDO) excess can contribute to the development of elevated blood pressure, as well as cardiovascular–renal–metabolic disease [13-17]. Treatment with mineralocorticoid receptor (MR) antagonists to block the actions of ALDO has been shown to offer cardio-renal protection [18-23]. The relationship between statin therapy and ALDO is not well established. In 2 separate observational studies—1 study in individuals with hypertension and 1 study in individuals with Type 2 diabetes mellitus—our group showed that chronic statin therapy was associated with lower serum and urinary ALDO as compared to individuals not on statins [24]. The magnitude of effect on ALDO was dependent on the type of statin: lipophilic statins (eg, simvastatin, atorvastatin, fluvastatin, and lovastatin) showed a greater association with lower ALDO as compared to hydrophilic statins (eg, pravastatin and rosuvastatin). Additionally, in ex vivo studies using rodent adrenal zona glomerulosa (ZG) cells, our group showed that ZG cells preincubated with lipophilic statins had a blunted ALDO response to stimulation by angiotensin II (AngII) as compared to hydrophilic statins [24]. However, the impact of statin therapy on ALDO in humans is not fully elucidated.

In the current randomized study, our aim was to formally test the hypothesis that (1) simvastatin lowers AngII-stimulated ALDO as compared to placebo and (2) a lipophilic statin (simvastatin) has a greater ALDO-lowering effect as compared to a hydrophilic statin (pravastatin).

## Methods

### Study design

We conducted a double-blind, randomized, placebo-controlled clinical trial (NCT02871687) at Brigham and Women's Hospital (BWH) Center for Clinical Investigation (CCI), Boston, MA, United States, to assess the effect of 12-week statin therapy vs placebo on ALDO.

### Participants

Healthy adults aged 18 to 70 years were recruited using online advertisements and physical flyers. Inclusion criteria included low-density lipoprotein cholesterol (LDL-C) > 70 mg/dL, blood pressure < 140/90 mmHg and >90/50 mmHg, and BMI, 19 to 40 kg/m^2^. Eligible participants had a normal physical exam, electrocardiogram, and screening laboratory values for sodium, potassium, glucose, liver enzymes, hemoglobin A1c, thyroid-stimulating hormone, and hemoglobin and had an estimated glomerular filtration rate of >60 mL/min/1.73 m^2^. Exclusion criteria included prescription medications other than stable thyroid hormone replacement, current pregnancy or breastfeeding, alcohol intake > 12 oz per week, tobacco or recreational drug use, or prior use of statin therapy. All study procedures were approved by the Mass General Brigham Institutional Review Board, and all participants provided written signed informed consent to enroll in the study protocol.

### Study protocol

**Figure 1:**
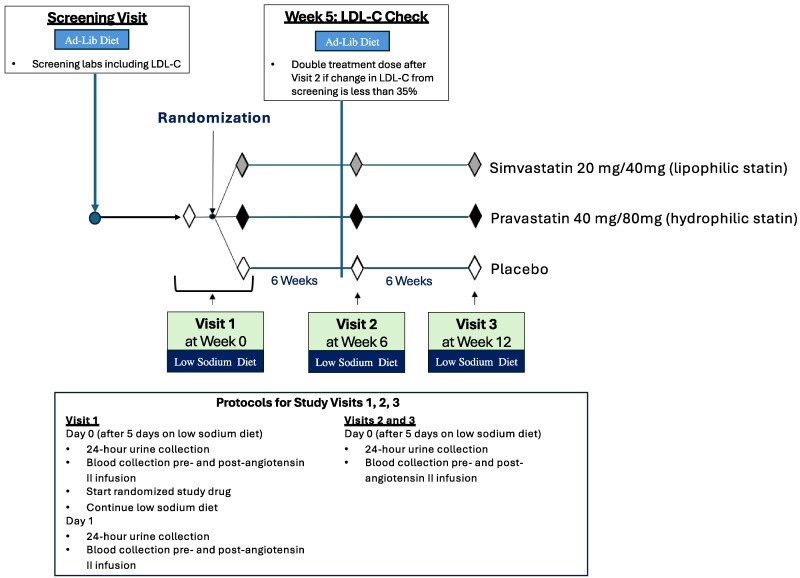
Study schema summarizing procedures at screening, Visit 1 (Week 0), Week 5, Visit 2 (Week 6), and Visit 3 (Week 12). Participants maintained an ad libitum (ad-lib) diet at screening and at Week 5. For Visits 1, 2, and 3, participants maintained a low-sodium diet for 5 days prior to the visit.

The detailed study protocol has been previously published [25]. Participants completed baseline (Visit 1), 6-week (Visit 2), and 12-week (Visit 3) overnight inpatient research admission visits for ALDO assessment (Fig. 1).

*Study diet:* To standardize ALDO stimulation, for 5 days prior to each visit, participants consumed a low-sodium (Na^+^), isocaloric diet (10 mEq Na^+^, 100 mEq potassium, and 100 mg calcium per day) provided by the BWH CCI Nutrition and Metabolic Research Core. A 24-hour urine collection was used to ensure compliance with the low-Na^+^ diet prior to conducting inpatient study procedures for each visit. Twenty-four-hour urine creatinine was used as an indicator of completeness of collection and urine analyte concentrations normalized to a full collection if urine creatinine was more than 15% above or below the individual's average 24-hour urine creatinine.

*Visit 1:* Participants were admitted to the CCI in the evening for a 2-night inpatient stay (Visit 1). Participants remained supine and fasted beginning at midnight. The next morning (Day 0), participants completed a prerandomization assessment consisting of a weight-based graded infusion of AngII (Giapreza, La Jolla Pharmaceutical, San Diego, CA) (30 minutes at 1 ng/kg/min followed by 30 minutes at 3 ng/kg/min) to stimulate ALDO secretion and enhance signal detection. Blood pressure was monitored at 2-minute intervals by trained licensed healthcare professionals during the infusion to ensure participant safety. Blood was collected via indwelling catheter for plasma renin activity (PRA), ALDO, and cortisol at baseline, 30 minutes, and 60 minutes of infusion, after which the infusion was stopped. Participants then received the first dose of the study drug to which they were randomized 1:1:1 (20 mg simvastatin, 40 mg pravastatin, or placebo), stratified by sex. Study staff and research participants were blinded to assigned treatments. Participants continued the low-Na^+^ diet, completed a second 24-hour urine collection throughout the day, and again remained supine and fasted beginning at midnight. The following morning (Day 1), participants received a second dose of the study drug and repeated the Day 0 studies (AngII infusion protocol) for their 1-day postinitiation drug therapy assessment. They were then discharged with study drug and instructed to take 1 pill every evening at home.

*Visit 2 (6 weeks)*: After 6 weeks of daily study drug, participants consumed a low-sodium diet for 5 days as described for Visit 1, 24-hour urine was collected, and participants were admitted to the CCI for 1 night (Visit 2) to repeat blood and AngII infusion procedures as performed on Day 0 at Visit 1.

Additionally, 1 week prior to Visit 2, blood was drawn to assess LDL-C level. Then following completion of the Visit 2 AngII infusion, study drug dose was doubled (40 mg simvastatin, 80 mg pravastatin, 0 mg placebo) if the LDL-C decreased by less than 35% from screening values.

*Visit 3 (12 weeks):* After a total of 12 weeks of daily study drug, participants were again admitted for 1 night to the CCI (Visit 3) and completed the same study procedures as Visit 2.

### Laboratory measurements

Serum ALDO, PRA, and cortisol as well as urinary ALDO and cortisol samples were analyzed at the Brigham Research Assay Core Lab at Brigham and Women's Hospital in Boston, Massachusetts. Serum ALDO (RRID: AB_2813725) and PRA (RRID: AB_3532145) were analyzed using enzyme-linked immunoassay; serum cortisol (RRID:AB_2802133) was analyzed using access chemiluminescent immunoassay; urinary ALDO (RRID: AB_2813725) was analyzed using enzyme-linked immunoassay; urinary cortisol was analyzed using liquid chromatography–mass spectrometry. Serum basic metabolic panel, serum insulin, lipid panel, and C-reactive protein as well as urinary creatinine samples were sent to Quest Diagnostics in Boston, Massachusetts. Urine sodium and potassium were picked up by the principal investigator and assayed using flame photometry as previously described [26].

### Statistical analyses

Continuous variables are expressed as mean ± standard deviation (SD), and categorical variables are presented as counts and percentages. To evaluate differences between the treatment groups, we employed 1-way ANOVA for continuous variables and either chi-square or Fisher's exact test for categorical variables. Post hoc comparisons were conducted using Tukey's test to identify specific group differences. Multivariate regression analyses were used to assess the primary endpoint, which was the change in AngII-stimulated ALDO from pretreatment to Week 12 posttreatment, controlling for sex, age, race, BMI, and pretreatment AngII-stimulated ALDO. Secondary endpoints, including differences in urinary ALDO and serum biomarkers, were analyzed using similar regression models. Normality assumptions for continuous variables were checked using the Shapiro–Wilk test. Nonparametric equivalents, such as the Kruskal–Wallis test, were applied for variables that violated these assumptions. Statistical significance was defined as *P* < .05. All statistical analyses were performed using SAS (version 9.4). Missing data were addressed using multiple imputation methods where applicable. Data analyses were conducted in adherence to an intention-to-treat principle, including all randomized participants with available data.

## Results

### Participant flow and pretreatment demographics

**Figure 2:**
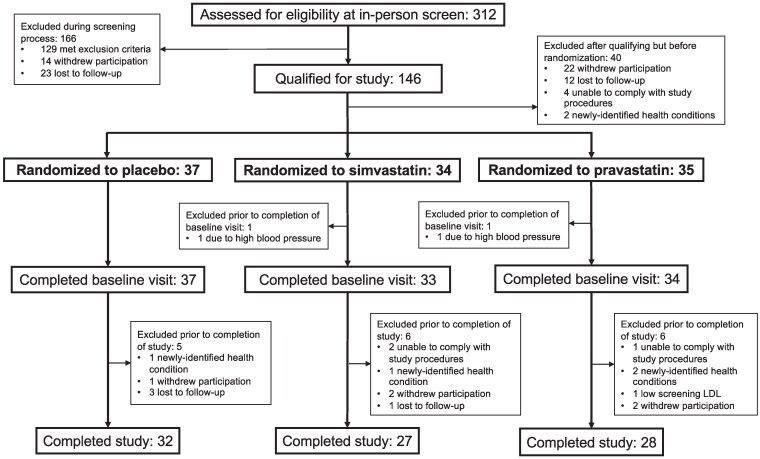
Participant flow diagram.

Of the 312 individuals who completed the screening visit, 106 were randomized to drug treatment (Fig. 2). Of these, 87 completed research procedures—32 randomized to placebo, 27 to simvastatin, and 28 to pravastatin. Demographics and pretreatment characteristics for these 87 individuals are shown in Table 1, separated by treatment arm; there were no significant differences between the 3 treatment groups at pretreatment. These were healthy adults with normal hemoglobin A1c, blood pressure, and without a history of clinical obesity [27].

**Table 1:** Demographics by treatment group

	Placebo (n = 32)	Simvastatin (n = 27)	Pravastatin (n = 28)	*P*
Age, years	35 ± 14	37 ± 11	33 ± 13	.63
Female, n (%)	14 (43.8)	12 (44.4)	13 (46.4)	.98
Race, n (%)	.51			
Asian/Pacific Islander	3 (9.4)	3 (11.1)	7 (25.0)	—
Black	3 (9.4)	4 (14.8)	1 (3.6)	—
White	24 (75.0)	18 (66.7)	17 (60.7)	—
More than 1/other	2 (6.3)	2 (7.4)	3 (10.7)	—
Latino or Hispanic, n (%)	1 (3.1)	1 (3.7)	5 (17.9)	.15
Body mass index, kg/m^2^	25.8 ± 4.2	27.2 ± 4.4	25.6 ± 3.8	.31
SBP, mmHg	120 ± 12	123 ± 9	118 ± 9	.22
DBP, mmHg	70 ± 9	74 ± 9	70 ± 9	.25
Hemoglobin A1c, %	5.2 ± 1.0	5.2 ± 0.3	5.3 ± 0.3	.96

Continuous data are presented as mean ± SD.

Abbreviations: DBP, diastolic blood pressure; SBP, systolic blood pressure.

### Dose escalation outcomes according to lipid response

As expected, the number of participants requiring dose escalation (those with a decrease in LDL-C < 35%) at 5 weeks was significantly different between treatment arms; 94% in the placebo group, 22% in the simvastatin group, and 50% in the pravastatin group were dose escalated, *P* < .01 compared to placebo (Table 2). At Visits 1, 2, and 3, lipids were similar in placebo, simvastatin, and pravastatin groups, as shown in Table 3. After 12 weeks of treatment (Visit 3), there was equivalent LDL-C lowering (% change from Visit 1 to Visit 3) in the pravastatin arm (−34.2%) and simvastatin arm (−39.5%), and these decreases were significantly different (*P* < .01) from that observed in the placebo treatment arm (+0.8%) (Table 3).

**Table 2:** Total cholesterol, triglycerides, HDL cholesterol, and LDL cholesterol (calc) were determined on ad-lib diets at screening and Week 5

		Placebo (n = 32)	Simvastatin (n = 27)	Pravastatin (n = 28)	*P_1_*	*P_2_*	*P_3_*
**Prerandomization**							
screening	Total cholesterol, mg/dL	185 ± 30	194 ± 40	185 ± 26	.38	.33	.91
	Triglycerides, mg/dL	98 ± 50	103 ± 54	92 ± 26	.73	.32	.54
	HDL cholesterol, mg/dL	57 ± 13	56 ± 11	60 ± 15	.80	.27	.38
	LDL cholesterol, mg/dL (calc)	109 ± 25	117 ± 34	106 ± 22	.31	.18	.64
**Postrandomization**							
Week 5 treatment dose check	Total cholesterol, mg/dL	183 ± 43	144 ± 47	154 ± 33	<.01	.23	<.01
	Triglycerides, mg/dL	120 ± 72	116 ± 99	105 ± 46	.85	.60	.33
	HDL cholesterol, mg/dL	57 ± 17	55 ± 19	61 ± 17	.69	.18	.30
	LDL cholesterol, mg/dL (calc)	102 ± 30	71 ± 30	72 ± 27	<.01	.82	<.01
	LDL cholesterol change screening to Week 5, %	−4.0 ± 16.5	−35.7 ± 23.3	−32.2 ± 18.4	<.01	.53	<.01
	Dose escalated, n (%)	30 (93.8)	6 (22.2)	14 (50.0)	<.01	.03	<.01

Study drug was dose escalated if there was less than a 35% decrease in LDL-C at Week 5 as compared to LDL-C at screening. Data are presented as mean ± SD. *P* values were determined by Student's *t* test, with the exception of chi-square for the number of individuals whose dose was escalated (*P_1_*, placebo vs simvastatin; *P_2_*, pravastatin vs simvastatin; *P_3_*, placebo vs pravastatin).

Abbreviations: HDL, high-density lipoprotein; LDL, low-density lipoprotein.

**Table 3:** Total cholesterol, triglycerides, HDL cholesterol, and LDL cholesterol (calc) were determined on a controlled low-sodium diet at Weeks 0, 6, and 12

		Placebo (n = 32)	Simvastatin (n = 27)	Pravastatin (n = 28)	*P_1_*	*P_2_*	*P_3_*
**Prerandomization**							
Visit 1	Total cholesterol, mg/dL	170 ± 29	177 ± 41	169 ± 30	.46	.40	.85
	Triglycerides, mg/dL	85 ± 45	95 ± 56	80 ± 37	.44	.22	.60
	HDL cholesterol, mg/dL	50 ± 12	47 ± 10	52 ± 14	.32	.14	.54
	LDL cholesterol, mg/dL (calc)	102 ± 26	110 ± 36	100 ± 27	.33	.23	.72
**Postrandomization**							
Visit 2 (Week 6)	Total cholesterol, mg/dL	168 ± 62	134 ± 55	134 ± 58	<.01	.96	<.01
	Triglycerides, mg/dL	87 ± 57	84 ± 55	66 ± 32	.82	.13	.08
	HDL cholesterol, mg/dL	50 ± 21	48 ± 18	53 ± 25	.60	.20	.43
	LDL cholesterol, mg/dL (calc)	100 ± 40	69 ± 35	67 ± 34	<.01	.86	<.01
Visit 3 (Week 12)	Total cholesterol, mg/dL	170 ± 24	130 ± 29	134 ± 31	<.01	.59	<.01
	Triglycerides, mg/dL	83 ± 45	83 ± 45	68 ± 22	.96	.14	.11
	HDL cholesterol, mg/dL	51 ± 13	48 ± 10	53 ± 18	.30	.18	.60
	LDL cholesterol, mg/dL (calc)	101 ± 21	65 ± 24	66 ± 27	<.01	.95	<.01
Change (Visit 1 to Visit 3)	Total cholesterol, %	0.4 ± 6.9	−25.5 ± 11.0	−20.3 ± 11.5	<.01	.09	<.01
	Triglycerides, %	0.4 ± 20.8	−10.6 ± 16.1	−9.1 ± 21.8	.03	.78	.09
	HDL cholesterol, %	1.4 ± 9.6	1.4 ± 10.8	1.2 ± 20.3	.98	.96	.95
	LDL cholesterol, %	0.8 ± 11.7	−39.5 ± 16.2	−34.2 ± 16.6	<.01	.23	<.01

Data are presented as mean ± SD. *P* values were determined by Student's *t* test, with the exception of chi-square for the number of individuals whose dose was escalated (*P_1_*, placebo vs simvastatin; *P_2_*, pravastatin vs simvastatin; *P_3_*, placebo vs pravastatin).

Abbreviations: HDL, high-density lipoprotein; LDL, low-density lipoprotein.

### Effect of study drug on aldosterone

Table 4 reports the serum and urine hormone results in participants consuming a controlled low-Na^+^ diet assessed at the following timepoints: Visit 1 (pretreatment and 1-day posttreatment initiation), Visit 2 (6 weeks postrandomization), and Visit 3 (12 weeks postrandomization). The pretreatment hormonal assessment from Visit 1 showed no statistically significant differences between treatment groups in 24-hour urine sodium, ALDO, and free cortisol and no statistically significant differences between treatment groups in serum ALDO, cortisol, and PRA assessed under upright posture, baseline supine conditions, and after AngII infusion.

**Table 4:** Blood and urine hormones were measured prerandomization to study drug and 1 day, 6 weeks, and 12 weeks postrandomization

		Placebo (n = 32)	Simvastatin (n = 27)	Pravastatin (n = 28)	*P_1_*	*P_2_*	*P_3_*
**Prerandomization**							
Visit 1 Day 0	Upright posture						
	Aldosterone, ng/dL	49.4 ± 17.4	44.6 ± 25.6	55.5 ± 37.2	.40	.22	.41
	Cortisol, ug/dL	11.9 ± 4.2	10.5 ± 4.3	10.6 ± 3.3	.21	.95	.19
	PRA, ng/mL/hr	8.9 ± 8.2	7.0 ± 5.2	7.3 ± 7.1	.30	.84	.43
	Supine baseline						
	Aldosterone, ng/dL	27.3 ± 12.9	24.7 ± 14.3	26.5 ± 20.2	.47	.71	.85
	Cortisol, ug/dL	10.8 ± 3.7	11.6 ± 3.6	11.3 ± 4.6	.41	.77	.67
	PRA, ng/mL/hr	2.8 ± 2.9	1.9 ± 1.5	2.4 ± 3.7	.18	.55	.65
	AngII-stimulated						
	Aldosterone, ng/dL	50.5 ± 17.4	41.4 ± 18.7	51.5 ± 31.4	.06	.15	.87
	Cortisol, ug/dL	9.2 ± 2.9	9.0 ± 4.1	9.2 ± 3.9	.87	.86	.97
	PRA, ng/mL/hr	1.2 ± 1.4	0.9 ± 0.7	0.9 ± 1.1	.23	.89	.33
	Urine						
	Sodium, mEq/24 hr	11.7 ± 7.9	15.1 ± 11.6	12.4 ± 9.4	.19	.33	.78
	Potassium, mEq/24 hr	70.6 ± 27.1	59.6 ± 18.4	67.0 ± 20.0	.08	.16	.57
	Aldosterone, ug/24 hr	33.3 ± 19.8	25.4 ± 13.4	25.4 ± 12.2	.08	0.99	.07
	Creatinine, mg/24 hr	1435.2 ± 432.5	1533.4 ± 568.5	1504.7 ± 635.3	.42	.84	.58
	Free cortisol, ug/24 hr	18.9 ± 23.8	18.3 ± 12.5	17.8 ± 11.3	.90	.88	.83
**Postrandomization**							
Visit 1 Day 1	Supine baseline						
	Aldosterone, ng/dL	28.0 ± 16.1	23.4 ± ± 13.1	25.7 ± 17.0	.25	.57	.61
	Cortisol, ug/dL	11.0 ± 3.6	11.6 ± 3.7	10.7 ± 3.8	.54	.36	.72
	PRA, ng/mL/hr	3.8 ± 6.3	2.2 ± 1.5	2.2 ± 2.1	.22	.95	.21
	AngII-stimulated						
	Aldosterone, ng/dL	49.6 ± 17.0	41.8 ± 15.7	52.5 ± 27.5	.07	.08	.62
	Cortisol, ug/dL	8.6 ± 2.5	9.1 ± 2.5	9.0 ± 2.7	.46	.85	.59
	PRA, ng/mL/hr	1.4 ± 2.1	0.9 ± 0.6	0.9 ± 0.9	.18	.93	.20
	Urine						
	Sodium, mEq/24 hr	12.3 ± 10.6	17.3 ± 9.7	15.2 ± 21.9	.07	.66	.51
	Potassium, mEq/24 hr	71.8 ± 27.8	68.3 ± 19.7	58.9 ± 20.4	.58	.09	.05
	Aldosterone, ug/24 hr	35.4 ± 22.5	20.9 ± 11.5	23.8 ± 15.2	<.01	.39	.02
	Creatinine, mg/24 hr	1475.5 ± 437.6	1571.3 ± 567.2	1499.9 ± 582.9	.43	.61	.84
	Free cortisol, ug/24 hr	19.3 ± 10.7	20.7 ± 17.1	20.5 ± 15.2	.70	.95	.73
Visit 2	Supine baseline						
	Aldosterone, ng/dL	25.2 ± 16.4	22.3 ± 15.1	22.5 ± 12.7	.48	.95	.47
	Cortisol, ug/dL	11.0 ± 4.6	11.2 ± 4.9	11.0 ± 5.3	.83	.82	.98
	PRA, ng/mL/hr	2.6 ± 3.3	2.0 ± 2.2	1.8 ± 1.7	.47	.64	.27
	AngII-stimulated						
	Aldosterone, ng/dL	44.4 ± 20.5	42.2 ± 23.2	51.5 ± 28.4	.65	.14	.18
	Cortisol, ug/dL	8.8 ± 3.8	10.1 ± 5.6	8.5 ± 4.3	.19	.17	.74
	PRA, ng/mL/hr	1.3 ± 2.2	0.9 ± 0.9	0.7 ± 0.7	.37	.53	.24
	Urine						
	Sodium, mEq/24 hr	15.0 ± 12.2	20.1 ± 15.2	20.7 ± 18.1	.18	.90	.19
	Potassium, mEq/24 hr	70.1 ± 30.7	63.4 ± 33.7	70.4 ± 42.0	.34	.46	.97
	Aldosterone, ug/24 hr	32.3 ± 18.0	21.1 ± 13.0	31.2 ± 38.9	<.01	.27	.90
	Creatinine, mg/24 hr	1455.1 ± 654.4	1467.6 ± 690.3	1397.4 ± 804.8	.92	.67	.70
	Free cortisol, ug/24 hr	16.0 ± 8.7	14.9 ± 8.0	23.4 ± 30.5	.58	.23	.25
Visit 3	Supine baseline						
	Aldosterone, ng/dL	24.7 ± 14.5	22.3 ± 15.1	24.5 ± 16.4	.54	.61	.95
	Cortisol, ug/dL	10.4 ± 3.5	10.1 ± 2.8	11.7 ± 4.0	.76	.10	.18
	PRA, ng/mL/hr	2.5 ± 2.2	1.8 ± 1.2	1.8 ± 2.1	.17	.97	.24
	AngII-stimulated						
	Aldosterone, ng/dL	49.6 ± 15.5	40.5 ± 21.2	47.3 ± 20.5	.06	.23	.62
	Cortisol, ug/dL	9.3 ± 4.3	9.2 ± 2.7	9.5 ± 3.3	.88	.70	.88
	PRA, ng/mL/hr	1.1 ± 1.0	0.8 ± 0.6	0.8 ± 1.0	.20	.98	.26
	Urine						
	Sodium, mEq/24 hr	13.9 ± 8.3	20.4 ± 18.2	15.2 ± 13.4	.07	.24	.64
	Potassium, mEq/24 hr	66.5 ± 22.7	58.6 ± 22.3	65.7 ± 27.1	.18	.29	.90
	Aldosterone, ug/24 hr	31.9 ± 15.4	22.3 ± 14.9	27.7 ± 17.6	.02	.22	.33
	Creatinine, mg/24 hr	1472.7 ± 457.2	1510.3 ± 561.8	1511.7 ± 580.2	.76	.99	.76
	Free cortisol, ug/24 hr	15.5 ± 7.4	16.9 ± 9.1	22.1 ± 16.6	.50	.15	.04
Change (Visit 1 Day 1 to Visit 3)	Supine baseline						
	Aldosterone, ng/dL	−2.6 ± 10.0	−2.4 ± 8.1	−2.0 ± 16.6	.94	.91	.87
	AngII-stimulated						
	Aldosterone, ng/dL	−0.9 ± 15.3	−2.0 ± 12.1	−4.3 ± 23.5	.77	.65	.50
	Urine						
	Aldosterone, ng/dL	−1.5 ± 15.7	−3.1 ± 11.6	2.4 ± 15.7	.66	.16	.36

Serum aldosterone, cortisol, and PRA were assessed following an upright posture study, supine prior to AngII infusion, and following 3 ng/kg/min AngII infusion. All assessments were done following completion of a low-Na^+^ diet. Data are presented as mean ± SD*. P* values were determined by Student's *t* test (*P_1_*, placebo vs simvastatin; *P_2_*, pravastatin vs simvastatin; *P_3_*, placebo vs pravastatin).

Abbreviations: AngII, angiotensin II; PRA, plasma renin activity.

**Figure 3:**
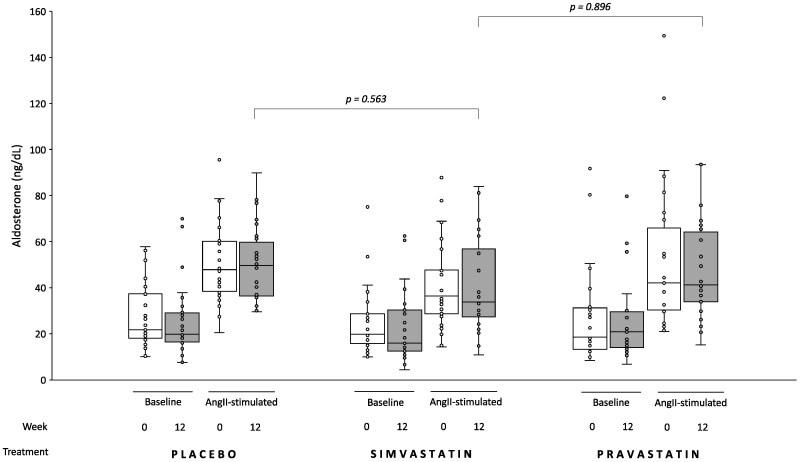
Comparison of baseline and AngII-stimulated aldosterone at Weeks 0 and 12 in placebo, simvastatin, and pravastatin treatment groups. Week 12 AngII-stimulated ALDO is compared between placebo and simvastatin groups (primary endpoint) and between simvastatin and pravastatin groups (secondary endpoint). *P* values are determined by multivariate regression analysis controlling for sex, age, race, BMI, and pretreatment AngII-stimulated ALDO.

To determine the impact of 12 weeks of treatment on our primary and secondary endpoints, AngII-stimulated ALDO on a low-Na^+^ diet, we performed a multivariate regression analysis controlling for our predetermined covariates of sex, age, race, BMI, and pretreatment AngII-stimulated ALDO. Twelve-week AngII-stimulated ALDO was similar between placebo and simvastatin (*P* = .563, primary endpoint) and between simvastatin and pravastatin (*P*, .896, secondary endpoint) (Fig. 3). The change in AngII-stimulated ALDO was similar between treatment groups, and there was no correlation between the change in LDL-C and the change in AngII-stimulated ALDO in either the simvastatin or pravastatin group. There was no effect of biological sex on outcome measures.

Twenty-four-hour urinary ALDO was consistently lower in those randomized to simvastatin compared to placebo throughout timepoints of the study: Visit 1, Visit 2, and Visit 3 (Table 4). However, given the slightly lower urine ALDO at pretreatment in the simvastatin group, when we assessed the change in urinary ALDO from Week 0 to Week 12, there was no statistically significant difference. There were also no differences in the change in serum ALDO or AngII-stimulated ALDO from Week 0 to Week 12 between treatment groups (Table 4).

## Discussion

In this randomized, placebo-controlled clinical trial of healthy adults without prior statin use, we did not find a reduction in serum or urinary ALDO on a low sodium diet after 12 weeks of treatment with statin therapy (simvastatin) vs placebo. Additionally, we did not find any significant difference in ALDO between treatment with a lipophilic statin (simvastatin) and treatment with a hydrophilic statin (pravastatin). Individuals treated with either simvastatin or pravastatin did have a clinically meaningful reduction of LDL-C levels after 12 weeks of treatment, whereas individuals treated with placebo did not show a decrease in LDL-C levels.

The findings of this study contrast with 2 observational studies—1 in individuals with hypertension and 1 in individuals with Type 2 diabetes mellitus—in which those receiving statins had lower serum and urinary ALDO as compared to those not on statins [24]. In the prior observational studies in humans, individuals were on statin therapy initiated by their prescribing physicians, presumably because they were thought to be at increased cardiometabolic risk. In the current trial, the study population included statin-naïve healthy adults, and individuals with known hypertension, diabetes, or coronary artery disease were excluded. The subject selection was intended to decrease the heterogeneity among participants, study a statin-naïve population, avoid ethical concerns regarding placebo administration in those who are clinically indicated for a statin, and minimize potential confounding variables in the results. However, given the overall cardiometabolic health of the subjects, it may be that the study population was too healthy to detect the impact of statins on ALDO. Additionally, the length of time potentially required for statins to impact ALDO remains unknown. In the prior observational studies in humans, individuals were on self-reported statin therapy for longer than 12 weeks on average [24]. Therefore, it is possible that an extended treatment period may be crucial to observe the pleiotropic effect of statins on ALDO.

Previous ex vivo studies showed that ZG cells isolated from rodent adrenal tissues and incubated for 15 minutes with statins produced lower ALDO and had a blunted response to AngII—suggesting an acute effect of statins on ALDO secretion by the adrenal glands [24]. In the current study, we did not observe an effect of statin therapy on ALDO production at any timepoint—1 day, 6 weeks, or 12 weeks. It may be that the mechanisms underlying the acute impact of statins on rodent adrenal glands differ from that on human adrenal glands. Additionally, the ex vivo studies showed that incubation with lipophilic statins had greater reduction of ALDO than incubation with hydrophilic statins. In the current study, we did not find any significant differences in the reduction of ALDO between treatment with simvastatin and pravastatin. The findings in the ex vivo studies may be explained by the direct effects that different statins had on ZG cells.

In this study, participants were studied on a low-sodium diet to amplify the ALDO to detect a potential reduction effect by statin therapy. Under normal physiology, a low-sodium diet stimulates the physiological adaptation of increasing AngII and increasing ALDO. However, statin treatment may not be potent enough to reduce ALDO in the presence of an activated renin–angiotensin–aldosterone system. It is possible that performing the study on a liberal sodium diet would have allowed us to see effects of statins on ALDO.

A limitation in the study is the difference in pretreatment ALDO between groups. Although the study was randomized, the pretreatment serum and urinary ALDO tended to be lower in the simvastatin group as compared to the pravastatin and placebo groups. While we corrected for pretreatment ALDO in the statistical analyses, the lower ALDO in the simvastatin group may have limited the magnitude of signal detection posttreatment. Additionally, although the current study did not detect significant differences, a larger sample size may be better powered to detect smaller effect sizes.

There are several strengths to this study. This was a rigorously designed, randomized, placebo-controlled clinical trial to study the impact of statins on ALDO. Healthy, statin-naïve participants were selected to minimize heterogeneity. Participants were studied under controlled dietary sodium, which is known to be an important regulator of ALDO. All study procedures were conducted in a highly controlled research environment. Participants were admitted and monitored in an inpatient research unit prior to any hormonal assessments, which were collected while patients were supine. Key variables known to impact ALDO secretion—including serum and urinary sodium, potassium, cortisol, and creatinine—were measured and compared across groups.

In conclusion, among a population of statin-naïve participants on a low-sodium diet, treatment with statin therapy was not associated with reduced ALDO when assessed after 1 day and Weeks 6 and 12 of treatment. However, given the healthy study population and study design, these results may not reflect the potential role of statins in reducing ALDO that was suggested by earlier observational studies. Understanding the pleiotropic impact of statins—one of the most commonly prescribed medications—in CVD risk reduction could have widespread public health implications. Therefore, additional research is needed to further elucidate the relationships between statin therapy, ALDO, and other potential mediators of CVD.

## Data Availability

Some or all datasets generated during and/or analyzed during the current study are not publicly available but are available from the corresponding author on reasonable request.

## Clinical trial information

NCT02871687.
